# The BRD4 Inhibitor I-BET-762 Reduces HO-1 Expression in Macrophages and the Pancreas of Mice

**DOI:** 10.3390/ijms25189985

**Published:** 2024-09-16

**Authors:** Ana S. Leal, Karen T. Liby

**Affiliations:** 1 Department of Medicine, Indiana University School of Medicine, Indianapolis, IN 46202, USA; anmendes@iu.edu; 2 Department of Pharmacology and Toxicology, Indiana University School of Medicine, Indianapolis, IN 46202, USA

**Keywords:** pancreatitis, macrophages, heme oxygenase, cancer prevention, bromodomain inhibitors

## Abstract

In pancreatic cancer, the tumor microenvironment (TME) accounts for up to 90% of the tumor mass. Pancreatitis, characterized by the increased infiltration of macrophages into the pancreas, is a known risk factor for pancreatic cancer. The NRF2 (nuclear factor erythroid 2-related factor 2) transcription factor regulates responses to oxidative stress and can promote cancer and chemoresistance. NRF2 also attenuates inflammation through the regulation of macrophage-specific genes. Heme oxygenase 1 (HO-1) is expressed by anti-inflammatory macrophages to degrade heme, and its expression is dependent on NRF2 translocation to the nucleus. In macrophages stimulated with conditioned media from pancreatic cancer cells, HO-1 protein levels increased, which correlated with higher NRF2 expression in the nuclear fraction. Significant differences in macrophage infiltration and HO-1 expression were detected in LSL-Kras^G12D/+^; Pdx-1-Cre (KC) mice, Nrf2 whole-body knockout (KO) mice and wildtype mice with pancreatitis. Since epigenetic modulation is a mechanism used by tumors to regulate the TME, using small molecules as epigenetic modulators to activate immune recognition is therapeutically desirable. When the bromodomain inhibitor I-BET-762 was used to treat macrophages or mice with pancreatitis, high levels of HO-1 were reduced. This study shows that bromodomain inhibitors can be used to prevent physiological responses to inflammation that promote tumorigenesis.

## 1. Introduction

Pancreatic cancer is the tenth and seventh most frequently diagnosed cancer in men and women, respectively. However, it is predicted to become the fourth and third most lethal cancer in the United States in men and women [1,2]. Deaths from this often rapidly lethal disease are predicted to surpass deaths from breast, prostate and colon cancer by 2030 [2], but in women, deaths from pancreatic cancer are expected to surpass colon cancer-related deaths this year [1]. Pancreatic cancer is often diagnosed at late stages, as most patients present with locally advanced (30–35%) or metastatic (50–55%) disease at diagnosis, with only 10–15% of patients able to undergo surgical resection of the tumor. Currently, the global 5-year survival rate for pancreatic cancer patients is 13%, but for patients that present at late stages or with metastatic disease, the survival rate at 5 years is only 1% [3,4,5]. Therapeutic approaches for the treatment of pancreatic cancer remain focused on chemotherapy, as several trials using targeted therapies and immunotherapies found no improvement over standard-of-care treatment for this disease [3,6,7,8].

The stromal reaction in pancreatic cancer can account for more than 50% of the tumor volume [9,10,11,12]. The stroma is partially responsible for therapeutic failure, both with conventional approaches and with new immunotherapeutic drugs [6,13,14,15]. Stromal components include non-transformed cells (inflammatory, immune, endothelial and mesenchymal stromal cells) and an extracellular matrix that promotes tumor progression, growth and metastasis, as well as alterations in the metabolic state of the tumor [9,16,17,18]. This complexity is intrinsic to pancreatic cancer and its poor prognosis. Because of the constantly evolving nature of the stromal compartment and its non-transformed status, epigenetic targeting drugs have been proposed for the treatment of pancreatic cancer [19,20,21,22,23,24].

Inflammation can promote pancreatic cancer, and pancreatitis, or inflammation of the pancreas, increases the probability of developing pancreatic cancer by 2.7–16.5-fold [25,26,27,28]. The regulation of multiple genes involved in inflammation and cell growth can be modulated by epigenetic modifications, more specifically by chromatin “readers” that detect and “read” specific modifications on the genome [29,30,31]. The bromodomain and extra terminal domain (BET) family of protein members read acetylated histones, thus regulating the assembly of chromatin complexes and transcription at specific promoter sites [32]. The BET family is composed of bromodomain-containing proteins: BRD2, BRD3, BRD4 and BRDT. BRD4, similar to other BET family members, contains two bromodomains that recognize acetylated lysine and is more often overexpressed in inflammation and cancer. Several small molecules have been designed to bind and inactivate BRD4, including JQ1 and I-BET-762, and are effective in reducing tumor growth and inflammation [31,33,34,35,36,37,38,39]. I-BET-762 was used in this study because of its favorable pharmacokinetics.

Macrophages are a heterogenous population of immune cells whose functions depend on the tissue location, the microenvironment and pathophysiological conditions. One of the main functions of macrophages is the regulation of immune homeostasis, and conventionally these cells can be described as having an M1 or M2 phenotype. M1 polarized macrophages promote inflammation via a Th1 inflammatory response, with the production of pro-inflammatory cytokines and reactive oxygen species (ROS). M2 polarized macrophages inhibit inflammation via a Th2 response by producing anti-inflammatory cytokines [40]. However, in pathological contexts like cancer, macrophages can exhibit mixed phenotypes that cannot be explained by the classical M1/M2 polarization. Most importantly, macrophages are highly plastic cells that can adapt to multiple conditions and express a multitude of different proteins, cytokines/chemokines and cell surface markers [41,42]. 

Heme, a complex of iron and protoporphyrin IX, plays critical functions in the survival of all aerobic organisms, including mammals and bacteria. Heme is usually associated with hemoglobin and myoglobin and is crucial for the transportation and storage of oxygen, respectively. Heme also associates with other proteins involved in the electron transfer of the respiratory chain, drug metabolism and oxidase and peroxidase enzymatic reactions. In humans, heme is present mainly in erythrocytes (80%) and the liver (15%). Recent findings also suggest that heme is essential for macrophage pathophysiological functions [43,44], such as the differentiation of monocytes to tissue-resident macrophages and/or in their activation by inflammatory stimuli. Heme oxygenase 1 (HO-1) is one of the enzymes expressed by classical anti-inflammatory macrophages and is also essential for regulating heme levels [43,44,45,46]. HO-1-deficient macrophages are sensitive to cell death after erythrocyte exposure. Erythrocyte heme is recycled in the spleen and liver and enzymatically degraded by HO-1 into equimolar amounts of carbon monoxide, iron and biliverdin. Manifestations of heme toxicity include excess ROS, lipid peroxidation, protein cross-linking and DNA damage. Because of the requirement of heme for cell maintenance, all cells have a stable cytoplasmic pool of heme.

The regulation of HO-1 is a complex process that involves the translocation of NRF2 into the nucleus. Upon oxidative or electrophilic stress or heme accumulation, the negative regulator of NRF2 (nuclear factor erythroid 2-related factor 2), KEAP1 (Kelch-like ECH-associated protein 1), is inactivated and targeted for proteasomal degradation while NRF2 translocates to the nucleus.

In the nucleus, NRF2 dimerizes with MAF (musculoaponeurotic fibrosarcoma) proteins, promoting the transcription of antioxidant target genes, including HO-1 [47,48]. In macrophages, NRF2 activation has anti-inflammatory effects [46,49], in contrast to its function in the epithelial cells of the pancreas, where NRF2 is essential for tumor progression and maintenance [50,51]. However, the role of NRF2-dependent HO-1 expression has never been investigated in the most prevalent immune cell found in pancreatic cancer, namely, the macrophage. Moreover, since NRF2 by itself is considered an undruggable target [52], we used a BRD4 inhibitor, I-BET-762, with anti-tumor properties in several cancers to suppress the activation of the NRF2 pathway. BRD4 is part of the NRF2 nuclear complex that activates downstream effectors, including HO-1 [53,54]. 

We hypothesized that an activating *Kras* mutation in the pancreas drives inflammation, which hijacks the physiological upregulation of HO-1 in macrophages, prolonging HO-1 expression and converting the physiological responses of macrophages for inflammation toward a tumor-promoting phenotype. Moreover, we tested the bromodomain inhibitor I-BET-762 in preventative intervention and to reduce HO-1 expression in macrophages. 

## 2. Results

### 2.1. Activation of the NRF2-HO-1 Pathway Correlates with Poor Survival in Patients with Pancreatic Cancer

**Figure 1:**
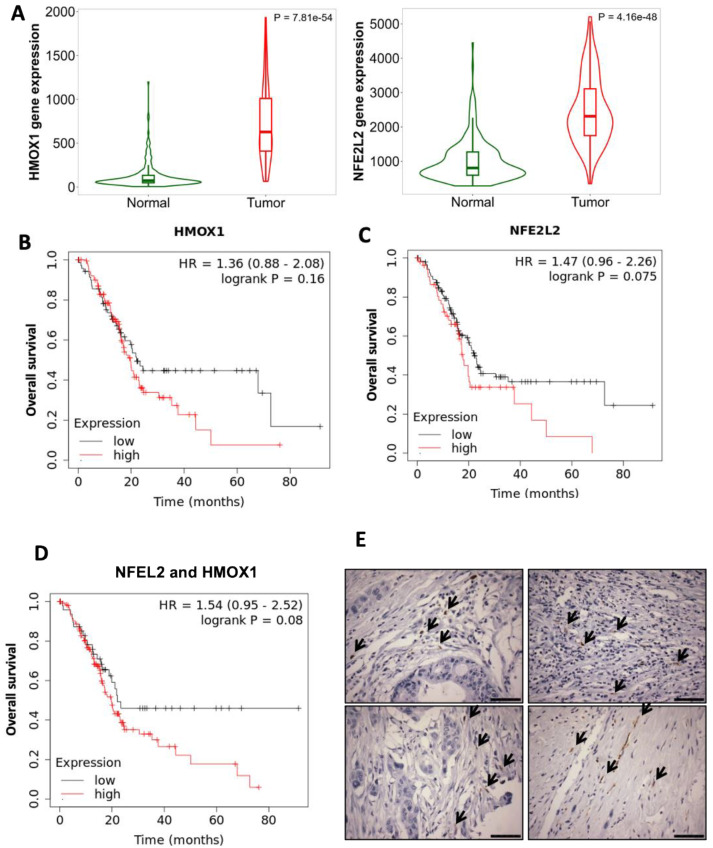
NRF2 (nuclear factor erythroid 2-related factor 2) and HO-1 (heme oxygenase 1) expression correlate with overall survival in patients with pancreatic cancer. (**A**) *HMOX-1* and *NFE2L2* (NRF2) expression was evaluated by RNA sequencing in tumors and adjacent normal tissue of patients with pancreatic cancer. Prognostic value of high vs. low gene expression of *HMOX1* (*n* = 73 high vs. 104 low) (**B**), *NFE2L2* (*n* = 123 high vs. 54 low) (**C**) and the combination (*n* = 50 high vs. 127 low) (**D**) in patients with pancreatic cancer (*n* = 177). Data for all plots were accessed and analyzed using KMPLOT Pan-Cancer RNA-seq data base, selected for pancreatic ductal adenocarcinoma. (**E**) Immunohistochemical evaluation of HO-1 expression in human pancreatic cancer samples from a tissue microarray; arrows indicate positive cells. Scale bar: 120 μm.

The gene encoding for NRF2, *NFE2L2*, is rarely mutated in pancreatic cancer. However, the activation of the NRF2 pathway leads to the increased expression of downstream effectors that promote tumorigenesis and tumor maintenance [50,51]. Heme oxygenase (HO-1 or the *HMOX1* gene in humans) is in part regulated by NRF2, and macrophages expressing HO-1 in pancreatic tumors can promote immunotolerance [55]. Analyzing data publicly available in KMplot [56], both *NFE2L2* and *HMOX1* expression are significantly (*p* = 4.16 × 10^−48^ and *p* = 7.81 × 10^−54^, respectively) upregulated in pancreatic adenocarcinomas compared with non-cancerous pancreatic tissue (Figure 1A). Additionally, low expression levels of *HMOX1* (Figure 1B) and *NFE2L2* (Figure 1C) correlate with extended survival in patients with pancreatic adenocarcinoma. Patients with low *HMOX1* expression had a median survival of 22 months, compared with an average of 19.7 months in patients with high expression. Similarly, median survival was 22 months in patients with low levels of *NFE2L2* compared to an average of only 17.7 months in patients with high *NFE2L2* expression. Combining *NFE2L2* and *HMOX1* (Figure 1D) expression also correlated with survival, as the cohort with low expression had a median survival of 22.8 months; the cohort with a high expression of both genes had a median survival of 19.7 months. High and low expression are automatically calculated by the cutoff values between the lower and upper quartiles of expression [56,57]. 

To validate the presence and location of HO-1 expression in human tumors, a tissue array of pancreatic adenocarcinomas (no normal pancreas was present in the array used) was stained for HO-1 (Figure 1E). HO-1 staining was observed in specific cells, at low percentages, within the matrix of the tumor, in accordance with a previous report [55].

### 2.2. Conditioned Media from Pancreatic Cancer Cells Increases Expression of HO-1 in Macrophages

**Figure 2:**
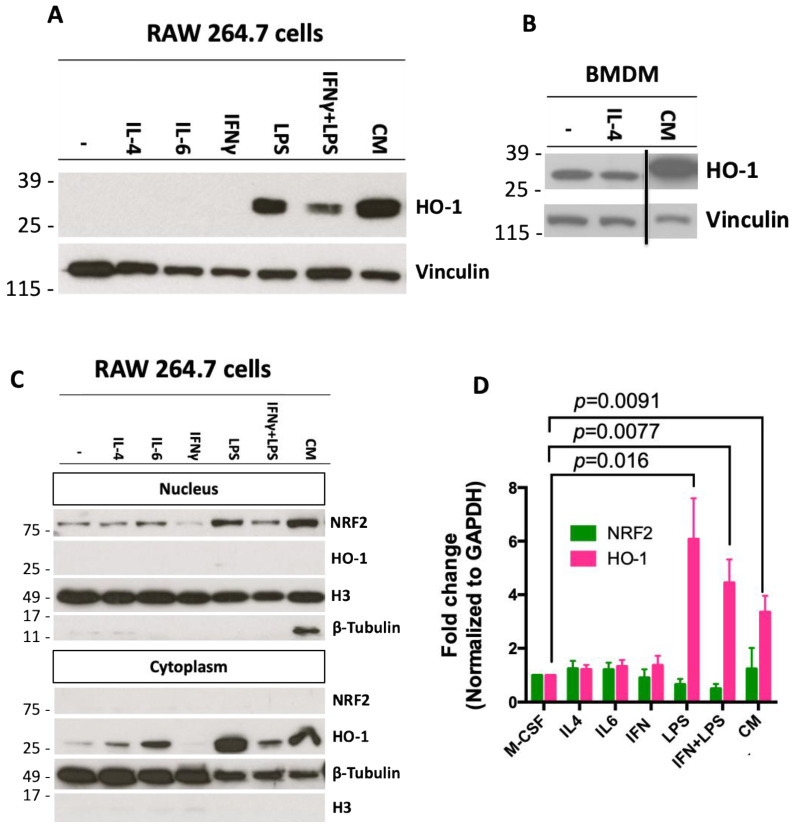
Inflammatory stimuli and secreted factors from cancer cells increase expression of HO-1 protein in macrophages. (**A**) Conditioned media (CM) from PanAsc 2159 cells was obtained by culturing cells at 70% confluence for 24 h. RAW 264.7 macrophage-like cells were treated with IL-4, IL-6, LPS and/or IFNγ at 10 ng/mL or CM for 24 h. (**B**) Bone marrow-derived macrophages (BMDMs) were stimulated with M-CSF for 5 days, followed by IL-4 or CM for 24 h. The Western blot was cut for simplification; the full blot is shown in Supplementary Figure S1A. (**C**) RAW264.7 cells were treated as described in panel A. Cytoplasmic and nuclear fractions were separated. All samples in panels (**A**–**C**) were analyzed by Western blotting. (**D**) mRNA levels of NRF2 and HO-1 in BMDMs cultured, as described in panel (**B**).

Macrophages expressing HO-1 can enable the progression of pancreatic cancer and tumor immunosuppressive properties [55]. To test if pancreatic cancer cells can elevate HO-1 in macrophages, RAW 264.7 macrophage-like cells and bone marrow-derived macrophages (BMDMs) were treated with conditioned media (CM) from pancreatic cancer cells. The PanAsc 2159 cancer cell line was derived from a LSL-Kras^G12D/+^; LSL-Trp53^R172H/+^; Pdx-1-Cre (KPC) mouse, a frequently used model of pancreatic cancer [58]. PanAsc 2159 cells were cultured for 24 h in media containing 1% FBS, and the media was harvested as CM. RAW 264.7 or BMDM cells were treated with several stimuli (all at 10 ng/mL) as controls in addition to the CM. Historically, IL-4 stimulation induces a M2 phenotype in macrophages, IL-6 activates the NRF2 pathway in macrophages [46], LPS and IFNγ activate M1 macrophages [59] and LPS is a known inducer of HO-1 [43]. BMDMs were collected from C57BL/6 mice and cultured for 5 days with M-CSF (10 ng/mL) to differentiate monocytes into macrophages. The media in the BMDMs was replaced by CM generated from PanAsc 2159 cancer cells or media containing IL-4. LPS, as expected, increased the expression of HO-1 in RAW 264.7 cells (Figure 2A). The CM elevated HO-1 expression in both RAW 264.7 cells (Figure 2A) and BMDMs (Figure 2B, Supplementary Figure S1A shows full Western blot). When proteins from RAW 264.7 cells were fractionated into nuclear and cytoplasmic portions, the increase in NRF2 in the nuclear fraction of the samples treated with LPS and CM corresponded to the increase in HO-1 in the cytoplasmic fraction (Figure 2C). Differences observed in the levels of HO-1 expression in Figure 2A,C for the IL-4 and IL-6 treatments are likely due to variations between the experiments and the enhanced sensitivity in the cytoplasmic extracts. In BMDMs, LPS, LPS + IFNγ and CM increased HO-1 mRNA expression by 6.1 (*p* = 0.0091), 4.4 (*p* = 0.0077)- and 3.3 (*p* = 0.016)-fold, respectively, compared to BMDMs treated with M-CSF alone (Figure 2D). No changes in the levels of NRF2 mRNA were identified in BMDMs with any of the treatments.

### 2.3. NRF2 Depletion Protects against Caerulein-Induced Pancreatic Lesions

**Figure 3:**
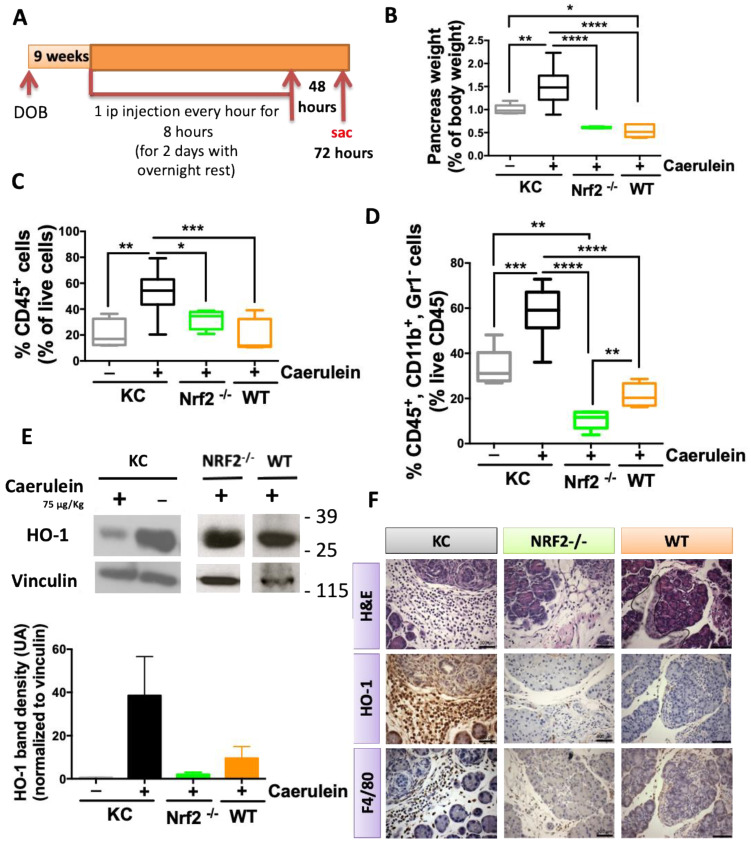
Increased expression of HO-1 in the pancreas of LSL-Kras^G12D/+^; Pdx-1-Cre (KC) mice. (**A**) Experimental design. Nine-week-old LSL-Kras^G12D/+^; Pdx-1-Cre (KC), wildtype (WT) or NRF2^−/−^ (knockout) mice were stimulated with caerulein (75 μg/Kg) to induce pancreatitis, and the pancreases were collected 72 h later, *n* = 5–10 mice/cohort. (**B**) Weight of pancreas at time of necropsy. (**C**) Percentages of CD45+ immune cells and (**D**) CD45+, CD11b+ macrophages in the pancreas were analyzed by flow cytometry. (**E**) HO-1 protein levels were determined by Western blot of total pancreas homogenates. (**F**) Immunohistochemistry was used to confirm the levels of HO-1 and the spatial localization of macrophages (F4/80) in the pancreas. Scale bar: 120 μm. * *p* ≤ 0.05, ** *p* ≤ 0.01, *** *p* ≤ 0.001, **** *p* ≤ 0.0001.

To determine if inflammation induced by pancreatitis also stimulates HO-1 expression in the pancreas, the 10-amino-acid oligopeptide caerulein was used to induce pancreatitis. Caerulein is an analog of cholecystokinin and gastrin that stimulates exocrine and proteolytic secretion in the pancreas, which consequently induces a fibroinflammatory reaction or pancreatitis. In humans, pancreatitis is associated with an increased risk of pancreatic cancer development [26,27]. Caerulein is commonly used to promote cancer progression in mouse models of pancreatic cancer [60,61]; however, these models do not evolve to form pancreatic tumors over their life spam. To evaluate HO-1 expression in the pancreas, LSL-Kras^G12D/+^; Pdx-1-Cre (KC), wildtype (WT) and whole-body Nrf2 knockout (Nrf2^−/−^) mice were used. Caerulein was injected ip every hour for 8 h at 75 μg/Kg, for 2 days with an overnight rest (Figure 3A) into 9-week old (adult) mice. A cohort of KC mice was also injected with saline at the same frequency as that of the mice injected with caerulein. Mice were sacrificed 48 h after the last day of injections and their pancreases were collected.

Pancreas weights were significantly (*p* < 0.0001) higher in KC mice stimulated with caerulein compared to WT and Nrf2^−/−^ mice. Additionally, pancreas weights in KC mice stimulated with caerulein were significantly (*p* < 0.01) higher than those injected with saline. The pancreases of WT mice (0.54% body weight, *p* < 0.05) and Nrf2^−/−^ (0.61% of body weight) mice weighed less than those of KC mice stimulated with saline (1% of body weight) (Figure 3B). Notably, the differences in weight paralleled the differences observed in total immune cell (CD45+) infiltration into the pancreas (Figure 3C). Infiltration of CD45 immune cells was significantly (*p* < 0.001) higher in KC mice stimulated with caerulein (52%) than in KC mice stimulated with saline (21.3%). KC mice stimulated with caerulein also had a significantly higher percentage of CD45 cells in the pancreas compared to WT (19.7%, *p* < 0.001) or Nrf2^−/−^ (31.7%, *p* < 0.05) mice stimulated with caerulein. 

Because disease progression in KC mice correlates with macrophage infiltration into the pancreas [62,63], we analyzed the percentage of macrophages infiltrating the pancreases of KC, WT and Nrf2^−/−^ mice (Figure 3D). In concordance with the changes observed for total immune cells, KC mice had the highest percentage of macrophages infiltrating into their pancreas. KC mice stimulated with caerulein had significantly (*p* < 0.001) higher numbers of macrophages (58.1%) than KC mice stimulated with saline (33.5%). WT (21.5%) and Nrf2^−/−^ (10.7%) mice had significantly (*p* < 0.0001) lower percentages of infiltration of macrophages when compared with KC mice stimulated with caerulein. Additionally, KC mice injected with saline had significantly (*p* < 0.01) higher infiltration percentages of macrophages compared to Nrf2^−/−^ mice stimulated with caerulein. Moreover, a significantly (*p* < 0.01) higher percentage of infiltrating macrophages was found in WT mice compared to Nrf2^−/−^ mice. Changes in macrophage infiltration detected by flow cytometry were confirmed by similar patterns in F4/80 immunostaining (Figure 3F).

Total protein extracts from the pancreases of KC, WT and Nrf2^-/-^ mice were also analyzed by Western blotting. KC mice stimulated with caerulein showed the largest increase in the expression of HO-1, followed by WT and lastly Nrf2^−/−^ mice (Figure 3E, Supplementary Figure S1B). Immunohistochemical analysis revealed that HO-1 expression was mainly concentrated in the stroma of the pancreatic tissue, similar to the F4/80 staining (Figure 3F).

### 2.4. The Bromodomain Inhibitor I-BET-762 Reduces HO-1 Expression Induced by Caerulein in the Pancreas of KC Mice

We and others have shown that small molecule inhibitors of BRD4 can reduce inflammation and disease progression in models of pancreatic cancer [39,64,65,66]. Moreover, BRD4 transcriptionally regulates the NRF2/HO-1 pathway in multiple disease settings when oxidative stress is upregulated [53,54]. Therefore, we hypothesized that the BRD4 inhibitor I-BET-762 could reduce HO-1 expression and, consequently, reduce the tumor-promoting polarization of macrophages in KC mice stimulated with caerulein. 

**Figure 4:**
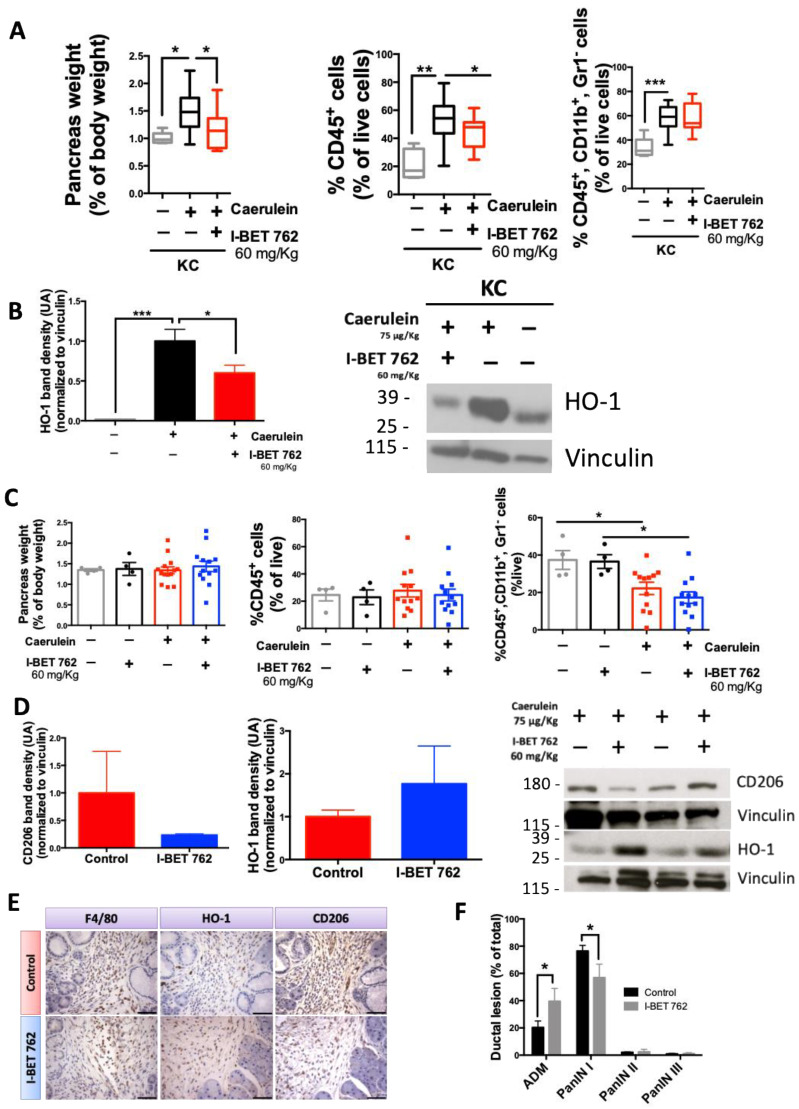
I-BET-762 reduced HO-1 and CD206 in the pancreas of KC mice. KC mice were stimulated with caerulein, as described in Figure 3. I-BET-762 diet was started 72 h before caerulein stimulation. Mice were euthanized 72 hours (**A**,**B**) or 9 weeks (**C**–**E**) after caerulein stimulation. Pancreas weights, levels of CD45+ immune cells and CD45+, CD11b+ macrophages in the pancreas were analyzed by flow cytometry 72 h (**A**) or 9 weeks (**C**) after caerulein stimulation. Levels of HO-1 and/or CD206 were determined by Western blot in total homogenates of pancreases treated for 72 h (**B**) or 9 weeks (**D**) with I-BET-762. (**E**) Immunohistochemistry was used to confirm the levels of HO-1 and CD206 expression and macrophage (F4/80) localization in the pancreases of KC mice 9 weeks after caerulein stimulation. Scale bar: 120 μm. (**F**) Quantification of acinar-to-ductal metaplasia (ADM) and pancreatic intraepithelial neoplasia (PanIN) lesions in the pancreas of KC mice 9 weeks after caerulein stimulation, *n* = 4/group. * *p* ≤ 0.05, ** *p* ≤ 0.01, *** *p* ≤ 0.001.

Using the same cohort of KC mice as in Figure 3, stimulated with saline or caerulein, an additional group of mice stimulated with caerulein and treated with I-BET 762 was added. The pancreas weight of the KC mice stimulated with caerulein (Figure 4A) and treated with I-BET-762 (60 mg/Kg diet) was significantly (*p* < 0.05) reduced compared to the KC mice treated with a control diet. Their reduction in pancreas weight was similar to KC mice stimulated with saline and receiving a control diet (1% vs. 1.2%). Total immune cell infiltration also was significantly (*p* < 0.05) reduced in KC mice treated with I-BET-762. However, no changes were observed in the percentage of macrophages infiltrating into the pancreas between KC mice receiving a control diet or I-BET-762. Despite a lack of differences in macrophage infiltration, levels of HO-1 protein were significantly (*p* < 0.05) reduced in KC mice stimulated with caerulein and treated with I-BET-762 compared to mice treated with a control diet and stimulated with caerulein (Figure 4B). In BMDMs, HO-1 mRNA expression was also decreased by I-BET-762 (Supplementary Figure S1C). In RAW 264.7 macrophage-like cells, I-BET-762 reduced the accumulation of Nrf2 in the nucleus, but no changes were observed at the RNA level (Supplementary Figure S1C,E). 

Pancreatic disease progresses in KC mice stimulated with caerulein over time, with increased fibrosis and higher-grade pancreatic cancer lesions [60,61]. Using the same caerulein protocol as in Figure 3A, KC mice were stimulated with caerulein or saline and treated with I-BET-762 or a control diet. Mice were sacrificed 9 weeks after caerulein or saline injections. Pancreas weights showed no differences between groups, with no changes observed in total percentage of immune cells infiltrating into the pancreas. In contrast, macrophage infiltration was significantly (*p* < 0.05) lower in KC mice stimulated with caerulein (Figure 4C). Whole pancreas protein lysates (Figure 4D) showed a lower level of CD206 in mice stimulated with caerulein and treated with I-BET-762 compared to mice treated with the control diet (1 vs. 0.22 UA). CD206 was used as a marker of tumor-promoting macrophages [67]. A trend toward higher HO-1 expression was observed for mice treated with I-BET-762 and stimulated with caerulein (1 vs. 1.8 UA). Immunohistochemistry (Figure 4E) was used to confirm the results from flow cytometry and Western blotting, showing expression of HO-1, CD206 and F4/80 in the stroma of the pancreas. 

A reduction in tumor-promoting macrophage markers such as Arginase 1 (Arg 1) and CCL2 [67] was also observed in BMDMs stimulated with conditioned media from pancreatic cancer cells and treated with I-BET-762 (Supplementary Figure S1D). Arg 1 was significantly (*p* = 0.0002) increased by conditioned media and significantly (*p* = 0.0002) reduced by I-BET 762 treatment. Analysis by a multiplex assay (Supplementary Figure S2) of selected samples of pancreas lysates 9 weeks post-caerulein in KC mice showed an overall decrease in the expression of cytokines and chemokines in mice treated with I-BET-762. Of special relevance were the observed reductions in the expression of IL-4 and M-CSF, known promoters of macrophage differentiation and infiltration with pro-tumorigenic properties [68,69]. 

The reduction in the expression of CD206 was accompanied with a significant (*p* < 0.05) reduction in the number of pancreatic intraepithelial neoplasia (PanIN) type I lesions in the pancreas of mice treated with I-BET-762 (Figure 4F). PanIN lesions are precursor lesions of pancreatic cancer that are graded from I to III, from low to high severity. Pancreatic acinar-to-ductal metaplasia (ADM) lesions are associated with inflammation, and when inflammation persists or *Kras* is mutated, these lesions evolve into PanIN [70]. In contrast to PanIN lesions, ADM lesions can be reversed, allowing acinar regeneration. The number of ADM lesions was higher in the pancreas of mice stimulated with caerulein and treated with I-BET-762 (Figure 4F), suggesting that I-BET-762 may interfere with the progression of ADM to PanIN.

### 2.5. Bromodomain Inhibitor I-BET-762 Prevents HO-1 Upregulation in the Pancreas of KPC Mice

HO-1-positive macrophages can reduce therapeutic responses in mouse models of pancreatic cancer and contribute to metastatic spread [55]. Because KC mice do not progress to pancreatic cancer and are mainly used to study inflammation in the context of *Kras* mutations, LSL-Kras^G12D/+^; LSL-Trp53^R172H/+^; Pdx-1-Cre (KPC) mice were used to test if I-BET-762 can reduce the expression of HO-1 in the pancreas before tumor formation. KPC mice usually develop pancreatic cancer around 22 weeks of age [58], and a high infiltration percentage of macrophages promotes disease progression [71]. 

**Figure 5:**
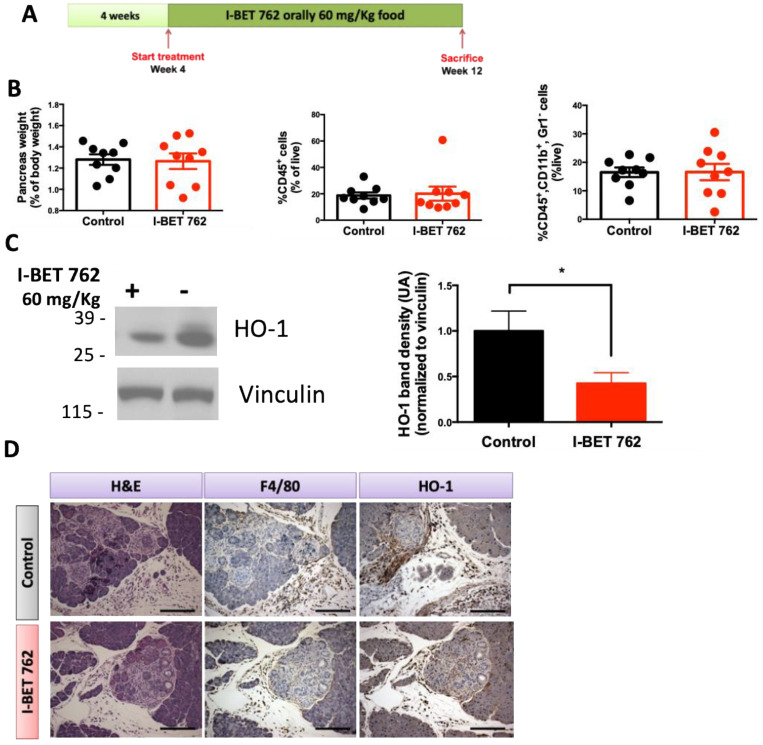
I-BET-762 reduced the expression of HO-1 in KPC mice. (**A**) Experimental design. LSL-Kras^G12D/+^; LSL-Trp53^R172H/+^; Pdx-1-Cre (KPC) mice were treated with I-BET-762 for 8 weeks, *n* = 9 mice/cohort. (**B**) Weight of pancreas, percentages of CD45^+^ immune cells and CD45^+^, CD11b^+^ macrophages were analyzed by flow cytometry in the pancreas. (**C**) Protein expression of HO-1 was determined by Western blot in total homogenates of pancreas, *n* = 9. (**D**) Immunohistochemistry was used to confirm expression of HO-1 and spatial localization of macrophages (F4/80) in the pancreas. Scale bar: 120 μm. * *p* ≤ 0.05.

KPC mice were started on a control diet or I-BET-762 diet (60 mg/Kg) at 4 weeks of age and maintained on the diet for 8 weeks. At 12 weeks of age, mice were sacrificed, and their pancreases were analyzed for immune cell infiltration and HO-1 expression (Figure 5A). At the time of sacrifice, no differences were observed in pancreas weight, total immune cell infiltration or macrophage infiltration between the control and I-BET-762 treatment groups (Figure 5B). Treatment with I-BET-762 significantly (*p* < 0.05) reduced HO-1 protein in whole pancreas lysates (Figure 5C). As observed in KC mice, HO-1 expression in the pancreas of KPC mice was mostly concentrated in the stroma of the pancreas, as observed by immunohistochemistry (Figure 5D). 

## 3. Discussion

Macrophages play important roles not only in pancreatic cancer progression [71,72] but also in pancreatitis [73]. Pancreatitis is a known risk factor for the development of pancreatic cancer [24,27,28]. Challenging KC mice with caerulein stimulates pancreatitis, which evolves into the development of progressive advanced pancreatic intraepithelial neoplasia (PanIN) over time, but rarely develops into fully advanced adenocarcinomas [60,61]. In pancreatitis, HO-1 upregulation in macrophages is part of the process required for the resolution of inflammation [43,44,74,75]. However, increased HO-1 expression (Figure 1) is correlated with cancer progression and with poor prognosis in pancreatic cancer [44,76,77,78]. We hypothesized that *Kras* mutations in epithelial cells disrupt the normal regulation of HO-1 in macrophages to resolve inflammation in pancreatitis, thereby prolonging HO-1 overexpression and therefore contributing to disease progression. 

This study shows that HO-1 elevation in the pancreas is greater when a *Kras* mutation is present (KC mice) and lower when Nrf2 is absent (Figure 3). HO-1 expression is partially regulated by NRF2 translocation to the nucleus and the consequent activation of downstream effectors [79]. NRF2 nuclear translocation occurs in stress conditions, such as pancreatitis. In normal conditions, increased HO-1 expression is required for the resolution of inflammatory processes. In clinical practice, several approaches have been tested to increase NRF2 translocation to the nucleus and, consequently, HO-1 upregulation in both macrophages and acinar cells [45,46,74]. 

NRF2 hyperactivation in the context of pancreatic cancer, either at the inception of disease or at the later stages, promotes disease progression [50,51]. However, most of these observations regarding NRF2 hyperactivation were made in acinar or epithelial cells that also carry an activating *KRAS* mutation, present in up to 95% of pancreatic cancers [80]. The work presented here shows that when *Kras* is mutated, HO-1 expression increases mainly in the stromal compartment of the pancreas, especially in the context of disease initiation by pancreatitis or in the LSL-Kras^G12D/+^; LSL-Trp53^R172H/+^; Pdx-1-Cre (KPC) genetic mouse model of pancreatic cancer. This physiological upregulation of HO-1, observed in macrophages, is hijacked by the *Kras* mutated cells, transforming the macrophages into CD206+ tumor-promoting macrophages (Figure 4E, Supplementary Figure S1). 

Epigenetic regulation is a comprehensive system that controls the progression and resolution of several inflammatory diseases, including pancreatitis [19,81,82]. Bromodomain inhibitors have been tested in inflammatory disease models and found to regulate immune cell functions and restore physiological properties [31,83,84]. Since pancreatitis is a known risk for pancreatic cancer, we hypothesized that the I-BET-762 bromodomain inhibitor could reduce HO-1 upregulation associated with pancreatitis. Moreover, bromodomains have been shown to be part of the NRF2 transcriptional complex [53,54] and are associated with pancreatitis [83,85], and inhibitors are effective in reducing tumor progression in mouse models of pancreatic cancer [65]. I-BET-762 treatment of BMDMs stimulated with conditioned media from pancreatic cancer cells reduced the expression of HO-1 and tumor-promoting markers, such as CCL2, CD206 and arginase 1 (Supplementary Figure S1). Similarly, the expression of HO-1 at early time points and CD206 at later time points (Figure 4) in the pancreas was reduced in KC mice treated with I-BET-762. Most importantly, I-BET-762 treatment reduced the number of PanIN lesions and increased ADM, reversible acinar-to-ductal metaplasia, lesions, suggesting that I-BET-762 can lead to acinar regeneration in the context of pancreatitis (Figure 4F). Additionally, in a preventative experiment, treatment with I-BET-762 also reduced the expression of HO-1 in the aggressive KPC murine model of pancreatic cancer (Figure 5). 

Targeting macrophages in cancer treatment has been pursued with varying results in preclinical models [86]. Since pancreatitis is a known risk factor for pancreatic cancer, targeting macrophages in this setting would be a likely option for the prevention of pancreatic cancer in high-risk patients [87]. Moreover, bromodomain inhibitors have shown promise in the treatment of pancreatitis and pancreatic cancer, both in murine models and in clinical trials [30,64]. HO-1 is a potential downstream target of bromodomain inhibitors, either because of its regulation of NRF2 transcription [53,54] or other mechanisms [88]. Additionally, not addressed in this manuscript is the possible reduction in NRF2 activation and, consequently, HO-1 expression in cancer cells by bromodomain inhibitors, since NRF2 has been shown to be essential for pancreatic cancer initiation and progression [50,51]. Therefore, based on this work, using bromodomain inhibitors to target the upregulation of HO-1 in pancreatitis, especially in patients with *KRAS* mutations or precursor lesions, might become a strategy to delay the initiation or progression of pancreatic cancer. 

## 4. Materials and Methods

### 4.1. Drugs

I-BET-762 was synthesized by J-Star Inc. (South Plainfield, NJ, USA) with purity > 95.

### 4.2. Cell Culture and Reagents

PanAsc 2159 cells were generated from KPC (LSL-Kras^G12D/+;^ LSL-Trp53^R172H/+^; Pdx-1-Cre) mice, as previously described [89], and grown in DMEM (Corning, Glendale, AZ, USA) with 10% FBS. RAW 264.7 mouse macrophage-like cells (ATCC, Manassas, VA, USA) were cultured in DMEM supplemented with 10% FBS. All cell lines were supplemented with 100 units/mL penicillin/streptomycin. Cells were cultured at 37 °C in a humidified incubator with 5% CO_2_. Bone marrow-derived macrophages (BMDMs) were obtained from wildtype (WT) or Nrf2^−/−^ mice; tibias and femurs were flushed with complete media and cells were plated with M-CSF (Biolegend, San Diego, CA, USA) at 10 ng/mL for 5 days. On day 5, media was replaced with fresh media containing the described chemokines/cytokines at 10 ng/mL or conditioned media from PanAsc2159 cells. 

### 4.3. In Vivo Experiments

All animal studies were conducted in accordance with protocols approved by the Institutional Animal Care and Use Committee at Michigan State University. KC mice (LSL-Kras^G12D/+^; Pdx-1-Cre) on a C57BL/6 background were obtained by interbreeding male LSL-Kras^G12D/+^; Pdx-1-Cre and female Pdx-1-Cre mice. KPC mice (LSL-Kras^G12D/+^; LSL-Trp53^R172H/+^; Pdx-1-Cre) on a C57BL/6 background were obtained by interbreeding male LSL-Kras^G12D/+^; Pdx-1-Cre and female Trp53^R172H/+^; Pdx-1-Cre mice. Genomic DNA was extracted from tail snips using the Extract-N-Amp tissue PCR kit (Sigma, St. Louis, MO, USA) and genotyped. Four-week old KC or KPC mice were randomized and fed a control 5002 diet or I-BET-762 in the 5002 diet (60 mg/kg diet); food was replaced twice a week [90]. To induce pancreatitis, KC mice were stimulated with saline (control) or caerulein (Sigma, St. Louis, MO, USA) at 75 μg/kg every hour for 8 h for 2 consecutive days, with an overnight rest. To reduce the number of animals used, the groups of KC mice (stimulated either with saline or caerulein) used for the 72 h studies depicted in Figure 3 and Figure 4 are the same cohort of mice. Experiments were performed simultaneously, but for clarification in the manuscript, they were divided into two figures. 

### 4.4. Flow Cytometry

One third of the pancreas and spleen removed from WT, Nrf2^−/−^, KC or KPC mice was minced and incubated separately in digestion DMEM media consisting of collagenase (300 U/mL, Sigma, St. Louis, MO, USA), dispase (1 U/mL, Worthington, Lakewood, NJ, USA) and DNAse (2 U/mL, Calbiochem, San Diego, CA, USA) for 30 min at 37 °C with stirring. The reaction was stopped by FBS addition, and cells were then passed through a 40 μm cell strainer (BD Falcon, Franklin Lakes, NJ, USA), and red blood cells were eliminated with 1X lysis solution (eBioscience, San Diego, CA, USA). Single cells were resuspended in a solution of PBS/0.5% BSA/0.1% azide and stained for 30 min at 4 °C with the antibodies CD45-VioGreen, Gr1-PE, CD11b-FITC (3 μg/mL, all Miltenyi, North Rhine-Westphalia, Germany) and 5 μg/mL anti-mouse CD16/CD32 antibodies (clone 93, eBioscience, San Diego, CA, USA) to reduce antibody binding to Fc receptors. Propidium iodide staining was used to exclude dead cells. Cells were analyzed using LSR II-DIVA 6.2 software (BD, Franklin Lakes, NJ, USA) with three laser sources (488 nm, 633 nm, 407 nm) and FlowJo V10.0.7r2 software (Trehe Star, Ashland, OR, USA).

### 4.5. Immunohistochemistry

One-third of the pancreas and spleen from mice was fixed in 10% phosphate-buffered formalin for at least 48 h, embedded in paraffin blocks and sectioned (5–6 μm). A solution of 3% hydrogen peroxide was used to quench endogenous peroxidase activity. Sections were immunostained with antibodies raised against CD206 (1:200, Abcam, Cambridge, UK), F4/80 (1:50, Abcam, Cambridge, UK) and HO-1 (1:500, Abcam, Cambridge, UK) and visualized with biotinylated anti-rabbit, anti-mouse or anti-rat secondary antibodies (Cell Signaling, Danvers, MA, USA, or Vector Labs, Newark, CA, USA). Signals were detected using a DAB substrate (Cell Signaling, Danvers, MA, USA), following the manufacturer’s recommendations. Sections were counterstained with hematoxylin (Vector Labs, Newark, CA, USA).

### 4.6. Western Blotting

For cells, RIPA buffer (1 M Tris-Cl, pH 7.4, 0.5 M EDTA, 5 M NaCl, 1% triton-X, 25 mM deoxycholic acid, 0.1% SDS) with protease inhibitors (PMSF, aprotinin and leupeptin) was used for lysis. Pancreases collected from the mice were homogenized in EBC buffer (1M Tris pH 8, 5 M NaCl) with the same protease inhibitors used in the RIPA buffer and 10% NP-40. Both the cell and pancreas samples were incubated on ice for 30 min. Protein concentrations were determined by the BCA assay (Sigma, St. Louis, MO, USA). Proteins were resolved by SDS-PAGE, transferred to a nitrocellulose membrane and analyzed with the following antibodies: vinculin (Cell Signaling, Danvers, MA, USA), HO-1, CD206 (Abcam, Cambridge, UK) and NRF2 (Proteintech, Rosemont, IL, USA). Secondary antibodies were purchased from Santa Cruz (Dallas, TX, USA) and Cell Signaling (Danvers, MA, USA). ImageJ (NIH, Bethesda, MD, USA) was used for the quantification of the immunoblots, and results were plotted and statistically analyzed using Prism 6. All images shown are representative of 2–3 independent experiments.

### 4.7. Multiplex Cytokine Assay

Cytokine levels in pancreas whole lysates were measured using a Millipore mouse 32plex kit (EMD Millipore, Burlington, MA, USA). Following the provided protocol, cytokines were measured using a Luminex^®^ 200TM array reader, and MAGPI^®^ (89-30000-00-236) software (Austin, TX, USA) with five-parametric-curve fitting was used for data analysis.

### 4.8. RT-PCR 

Cells were collected and RNA was extracted using TRIzol (Thermo Fisher, Waltham, MA, USA), following the manufacturer’s instructions. Two micrograms of RNA was reverse transcribed, and 2 µL of complementary DNA from this reaction was added to 5 µL of iQ SYBRGREEN Supermix (Bio-Rad, Hercules, CA, USA) and 1 µL of validated RT2 quantitative PCR (qPCR) primers: Nrf2 (Gene ID: 18024; F: 5′-GATCCGCCAGCTACTCCCAGGTTG-3′; R: 5′-CAGGGCAAGCGACTCATGGTCATC-3′), HO-1 (Gene ID: 15368; F: 5′-GAGCAGAACCAGCCTGAACTA-3′ R: 5′-GGTACAAGGAAGCCATCACCA-3′); CD206 (Gene ID: 17533; F: 5′CAGGTGTGGGCTCAGGTAGT 3′ R: 5′ TGTGGTGAGCTGAAAGGTGA 3′) Il-10 (Gene ID: 16153; F: 5′ GCTCTTACTGACTGGCATGAG 3′R: 5′ CGCAGCTCTAGGAGCATGTG 3′), iNOS (Gene ID: 18126; F:5′-GAGATTGGAGTTCGAGACTTC-3′, R:5′-TGGCTAGTGCTTCAGACTTC-3′), Arg1 (Gene ID: 11846; F: 5′-CTCCAAGCCAAAGTCCTTAGAG-3′, R: 5′-AGGAGCTGTCATTAGGGACATCGAPDH-3′), GAPDH (Gene ID: 14433; F: 5′-GGAGCGAGATCCCTCCAAAAT-3′; R: 5′-GGCTGTTGTCATACTTCTCATGG-3′).

### 4.9. Patient Survival Analysis

*HMOX1* (HO-1) and *NFE2L2* (NRF2) were used to analyze relapse-free survival and compare tumor vs. adjacent normal tissue. Aggregate data from KMPlot Pan-cancer RNA-seq (http://www.kmplot.com, accessed on 15 January 2024) were auto-selected for the best cutoff (25th and 75th percentiles), and selected pancreatic ductal adenocarcinomas included all stages, genders, races, grade, mutation burden and neoantigen load. Data analysis was not restricted based on cellular content [56,57]. A tissue array of human pancreatic cancer samples was purchased from TissueArray.com (Derwood, MD, USA) and analyzed for HO-1 expression by immunohistochemistry as described above. Ethical approval was not required due to the retrospective nature of this study. 

### 4.10. Statistical Analysis

Unless noted, all experiments were repeated at least three times, and representative images are shown. Results are described as mean ± standard error. Data were analyzed by a paired t-test with Welch’s correction for sets with two variables, or ordinary one-way analysis of variance followed by Dunnett’s multiple comparisons test (Prism 6). All *p* values are two-sided; *p* < 0.05 was considered statistically significant.

## Data Availability

Data for patient survival and relapse-free survival available at KMPLOT.com.

## Supplementary Materials

The following supporting information can be downloaded at https://www.mdpi.com/article/10.3390/ijms25189985/s1.
