# Supplementary material for: The BRD4 Inhibitor I-BET-762 Reduces HO-1 Expression in Macrophages and the Pancreas of Mice
Source: Int J Mol Sci. 2024 Sep 16;25(18):9985. doi: 10.3390/ijms25189985 (PMC11432103; doi:10.3390/ijms25189985)
Supplement: Supplementary file 1 [file ijms-25-09985-s001.zip › ijms-3179927-supplementary.pdf]

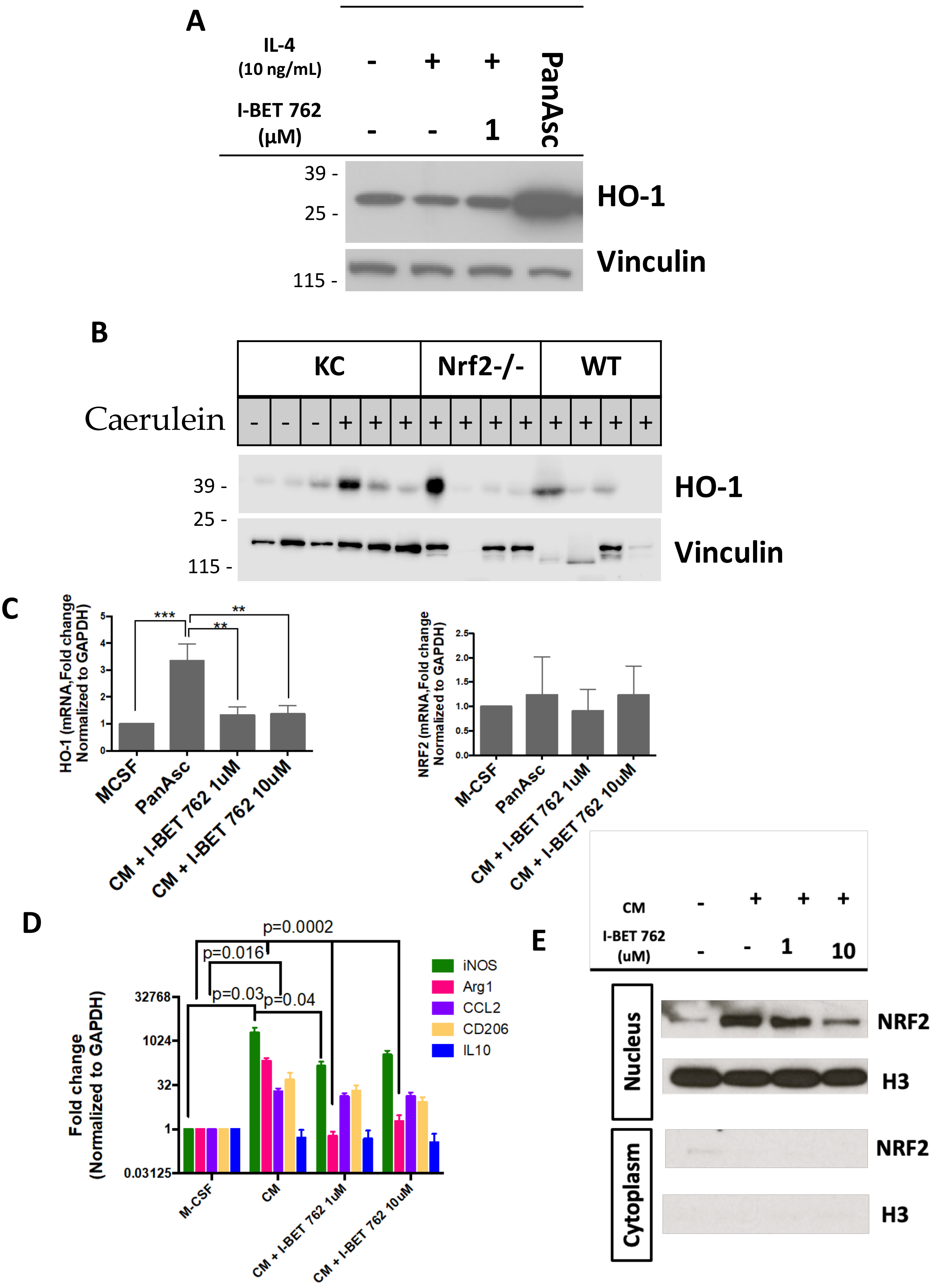

A
IL-4 (10 ng/mL)
- + + PanAsc
I-BET 762 (µM)
- - 1
39 -
25 -
115 -
HO-1
Vinculin
B
KC
Nrf2-/-
WT
Caerulein
- - - + + + + + + + + + + +
39 -
25 -
115 -
HO-1
Vinculin
C
HO-1 (mRNA,Fold change Normalized to GAPDH)
***
**
**
MCSF
PanAsc
CM + I-BET 762 1uM
CM + I-BET 762 10uM
NRF2 (mRNA,Fold change Normalized to GAPDH)
M-CSF
PanAsc
CM + I-BET 762 1uM
CM + I-BET 762 10uM
D
p=0.0002
p=0.016
p=0.03
p=0.04
Fold change (Normalized to GAPDH)
32768
1024
32
1
0.03125
iNOS
Arg1
CCL2
CD206
IL10
M-CSF
CM
CM + I-BET 762 1uM
CM + I-BET 762 10uM
E
CM - + + +
I-BET 762 (uM) - - 1 10
Nucleus
Cytoplasm
NRF2
H3
NRF2
H3

	Control			I-BET 762		
GCSF	30.59	6.47	95.25	5.1		
EOTAXIN	1815.85	112.20	34.87	1133.9	**49.7**	**24.5**
GM-CSF	41.08	43.78	18.50	65.4	26.4	22.6
IFNG	11.34	84.12	46.24	10.3	4.0	14.0
IL-1A	131.88	354.90	1236.85	168.6	127.5	106.6
IL-1B	20.34	43.83	52.89	33.9	21.4	36.5
IL-2	14.61	11.53	6.33	12.8	12.3	18.9
IL-4	142.03	6.08	151.31	0.6	0.7	0.7
IL-3	1.47	1.47	60.43	0.9	0.3	1.4
IL-5	5.52	2.02	2.48	2.5	0.9	0.5
IL-6	230.09	24.31	164.13	9.9	4.8	17.1
IL-7	10.99	44.38	12.33	15.4	10.6	11.8
IL-9	236.97	1235.20	37.70	377.1	232.1	293.3
IL-10	19.28	152.74	149.79	20.8	9.4	54.5
IL-12P40	27.47	20.92	68.24	18.6	14.6	44.7
IL-12P70	3.81	9.37	20.83	5.8	4.8	13.0
LIF	183.48	93.61	215.27	246.4	89.0	3.1
IL-13	112.36	524.03	1199.43	185.3	237.4	179.6
LIX	115.47	78.73	424.65	70.7	41.6	77.8
IL-15	83.29	485.11	96.95	149.2	97.0	87.4
IL-17	5.98	81.87	2075.19	3.2	1.4	1.0
IP-10	77.50	73.96	502.33	109.3	41.1	10.0
KC	1151.96	451.58	34.87	966.4	387.9	25.6
MCP-1	449.02	45.00	27.24	134.0	32.8	30.3
MIP-1A	230.96	72.87	159.19	64.2	68.4	75.5
MIP-1B	134.76	19.21	14.41	18.2	15.2	18.5
M-CSF	127.52	44.72	162.53	46.9	35.8	31.2
MIP-2	1443.62	217.48	189.78	146.9	117.2	112.2
MIG	115.22	150.54	67.67	287.8	124.5	13.5
RANTES	43.98	116.09	1101.55	29.0	20.0	14.8
VEGF	182.01	39.41	53.63	64.8	28.2	74.1
TNFA	6.43	10.41	4.55	4.2	4.6	5.5

Supplementary Figure S2. I-BET-762 reduced expression of cytokines and chemokines in the pancreas of KC mice treated with I-BET-762 for 9 weeks. LSL-Kras$^{G12D/+}$; Pdx-1-Cre (KC) mice were treated with I-BET-762 as described in Figure 4. Pancreas were homogenized in EBC buffer and analyzed in a luminex assay following the recommended protocol.
